# Artificial intelligence-, organoid-, and organ-on-chip-powered models to improve pre-clinical animal testing of vaccines and immunotherapeutics: potential, progress, and challenges

**DOI:** 10.3389/frai.2025.1681106

**Published:** 2025-10-31

**Authors:** Elhoucine Elfatimi, Yassir Lekbach, Swayam Prakash, Sweta Karan, Joshua Christian Dorotta, America Garcia, Beverly Sabathini Suoth, Chhaya Maurya, Etinosa Yvette Omorogieva, Sarah Xue Le Ng, Emma Jane Liao, Reilly Andrew Chow, Lbachir BenMohamed

**Affiliations:** ^1^Laboratory of Cellular and Molecular Immunology, College of Medicine, The Gavin Herbert Eye Institute, University of California, Irvine, Irvine, CA, United States; ^2^Institute for Immunology, University of California, Irvine, Irvine, CA, United States; ^3^Chao Family Comprehensive Cancer Center, University of California, Irvine Medical Center, Orange, CA, United States; ^4^Department of Vaccines and Immunotherapies, TechImmune, LLC, University Lab Partners, Irvine, CA, United States

**Keywords:** artificial intelligence—AI, organoid, organ-on-chip, immunotherapeutics, vaccines, infectious diseases, deep learning

## Abstract

Vaccines and immunotherapies against infectious diseases and cancers have been a great success of the medical sciences over the last century. Pre-clinical testing in animal models has played a crucial role in the development of vaccines and immunotherapies, informing subsequent clinical trials. The current practices in pre-clinical animal model research must be approved by committees with strict policies and assessments on animal experiments including the “three Rs”: (1) Replacement, which assesses the scientific justification and rationale for using a live animal in biomedical research; (2) Reduction, which determines whether the number of animals required in an experiment is adequate to achieve scientifically valid results while reducing costs; and (3) Refinement, which ascertains that any given animal procedure will cause no to minimal pain or distress. The recent initiatives by the United States NIH and FDA to reduce or phase out animal testing in biomedical research underscore a growing interest in artificial Intelligence (AI), deep learning (DL), organoid, and organ-on-chip-powered models to slash the time and cost of preclinical animal research. This review highlights the strengths, progress, and limitations of these alternative pre-clinical research approaches, with a focus on vaccine and immunotherapeutic development. While the implementation of AI- and DL-, organoid-, and organ-on-chip-powered models will certainly help accelerate pre-clinical discoveries, modeling the safety, immunogenicity, and protective efficacy of vaccines and immunotherapeutics as they occur *in vivo* is not yet comprehensive enough to fully replace or replicate the complexity of living systems, in both animals and humans. Thus, these models should be viewed as powerful complementary tools that combine hybrid human and artificial intelligence and must be validated through animal model testing. This review discusses the path forward and the scientific challenges that persist in investing in AI- and DL-human hybrid validation systems, regulatory reforms, and the development of interconnected platforms that bridge digital models with biological reality.

## 1 Introduction

Animal experimentation has long underpinned advances in immunology, vaccine development, and immunotherapeutic advancements, providing mechanistic insights into immune processes and offering platforms for pre-clinical testing of novel therapeutics (Quadiri et al., 2025a; Corleis et al., 2023; Tesfamariam et al., 2022). Traditionally, rodent and non-human primate models have been central to these efforts, enabling researchers to explore antigen processing, immune memory, and vaccine efficacy in controlled *in vivo* systems (Wang et al., 2021). While prior reviews have addressed organoids (Wagar, 2023; Kastenschmidt et al., 2023; Chen et al., 2021), artificial intelligence (AI) (Elfatimi et al., 2025), and organ-on-a-chip models (Jeger-Madiot et al., 2024; Shahabipour et al., 2023) separately, few have critically examined how these approaches converge to improve animal testing, specifically within the context of vaccine and immunotherapeutic development. This review aims to bridge the translational gap between pre-clinical animal studies and human clinical trials. We discuss the potential, progress, and challenges of AI-, organoid-, and organ-on-chip-powered models in improving pre-clinical testing of vaccines and immunotherapeutics.

Preclinical testing in animal models has led to major medical breakthroughs, including the early development of vaccines against smallpox and polio. However, in recent years, questions have intensified regarding the translational validity and ethical justification of continued reliance on animal testing, particularly when translating findings to human clinical outcomes (Rudroff, 2024; Willner, 1986). Lack of methodological rigor and statistical quality in preclinical animal research can impact the validity, reproducibility, and translational value of scientific findings (Deng and Strong, 2025; Han, 2025; Moassefi et al., 2023). These include (1) a lack of randomization and blinding of investigators, which introduces selection and detection biases that can distort results (Osborne et al., 2018); (2) lack of proper power calculations and sample size estimations, which can lead to studies that are underpowered and produce results with limited statistical confidence (Osborne et al., 2018); (3) not considering biological variables including gender differences that may reduce the applicability and generalizability of findings and may undermine the predictive value of animal models, which may contribute to a failure of clinical trials (Osborne et al., 2018; Gopel and Burggren, 2022; Gualtierotti, 2025). This calls for standardized reporting protocols, the mandatory incorporation of design elements such as randomization, blinding, and power calculations, as well as improved training to prioritize methodological quality and transparency (Osborne et al., 2018; Percie du Sert et al., 2020).

**Figure 1:**
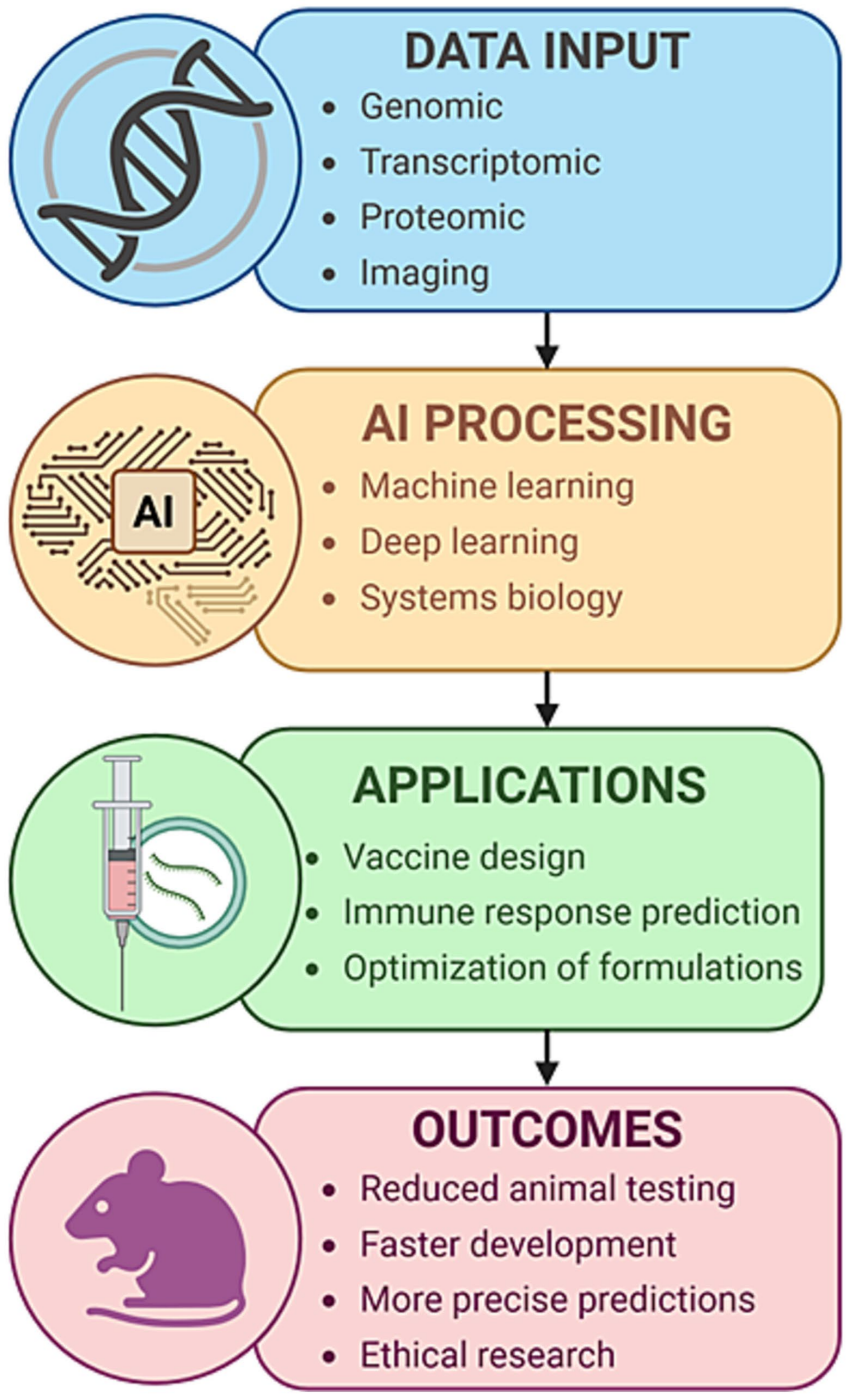
AI-based computational modeling in immunology and vaccine development. A conceptual overview of an AI-driven pipeline for accelerating immunology research and vaccine development. The workflow begins with multimodal biological inputs, including genomic, transcriptomic, proteomic, and imaging data derived from human-relevant sources. These data are processed using advanced artificial intelligence techniques, including machine learning, deep learning, and systems biology modeling. The outputs inform key applications, including vaccine design, prediction of immune responses, and formulation optimization. This process ultimately contributes to reduced animal testing, faster and more precise vaccine development, and the advancement of ethical biomedical research.

Structural and functional differences in immune cell repertoires, cytokine expression profiles, and pathogen recognition pathways between species often lead to misleading results. For example, promising immunotherapies and vaccines that succeed in murine models frequently fail in Phase I or II clinical trials, highlighting the limited fidelity of animal models in replicating human immune complexity (Willner, 1986). Moreover, animal research is associated with significant costs and logistical burdens, including the maintenance of specialized facilities, breeding colonies, and compliance with strict regulatory frameworks. Figure 1 illustrates an AI-enabled pipeline that integrates human-relevant biological data, including omics and imaging, into machine learning models to support vaccine design, predict immune responses, and assess toxicity (Elfatimi et al., 2025; Kleinstreuer and Hartung, 2024; Farzan, 2024; Sharma et al., 2022). This approach significantly reduces the need for animal models while promoting ethical and human-relevant biomedical research. Recent developments, such as *in silico* clinical trials, explainable AI, and digital immune twins, further expand the boundaries of what can be accomplished using human-based, AI-supported systems.

For instance, the AlphaFold 3 model represents a significant advancement in biomolecular structure prediction, accurately modeling protein monomers, protein multimers, and complex biomolecular assemblies that involve proteins, DNA, RNA, ligands, ions, and chemical modifications (Desai et al., 2024; Elfmann and Stulke, 2025). AlphaFold 3 uses a next-generation DL architecture with an improved Evoformer module and a novel diffusion network approach (Desai et al., 2024; Elfmann and Stulke, 2025). This diffusion process starts with a cloud of atoms. It iteratively refines the structure to converge on a highly accurate joint 3D model of the input molecules, enabling detailed insight into biomolecular interactions and complexes (Desai et al., 2024; Elfmann and Stulke, 2025). Thus, AlphaFold 3 represents a significant leap forward for structural biology, systems biology, and the discovery of antibody-mediated vaccines and immunotherapeutics, enabling accurate predictions of complex biomolecular assemblies within a single framework. This opens new avenues for biomedical research and therapeutic development. However, there are key limitations of AlphaFold 3 compared to other models, such as ZDOCK, especially in the context of protein–protein docking (Harmalkar et al., 2025; Abramson et al., 2024). Unlike ZDOCK and other similar physics-based docking approaches, AlphaFold 3 does not capture the dynamic conformational changes that occur during binding (Harmalkar et al., 2025; Abramson et al., 2024). Although AlphaFold 3 produces accurate protein structures, its docking orientation predictions for protein-antibody complexes can sometimes be incorrect, leading to flawed binding interfaces (Harmalkar et al., 2025; Abramson et al., 2024).

Nonetheless, these emerging platforms are not without limitations. AI models are only as good as the data on which they are trained, and biases in publicly available immunological datasets can skew predictions or mask relevant signals (Yang et al., 2024). Moreover, while organoids and chips can replicate localized responses, they do not yet fully model the integrated, systemic nature of immune reactions seen in living organisms. Therefore, although the long-term vision is to develop fully human-relevant pre-clinical pipelines, animal models continue to play a necessary, though diminishing, role, particularly in assessing safety, immunogenicity, and protective efficacy of vaccine and immunotherapeutic candidates by providing predictive computational models, identifying patterns of B- and T-cell response, and supplementing traditional *in vivo* animal pre-clinical trials (Elfatimi et al., 2025; Reveiz et al., 2025).

**Figure 2:**
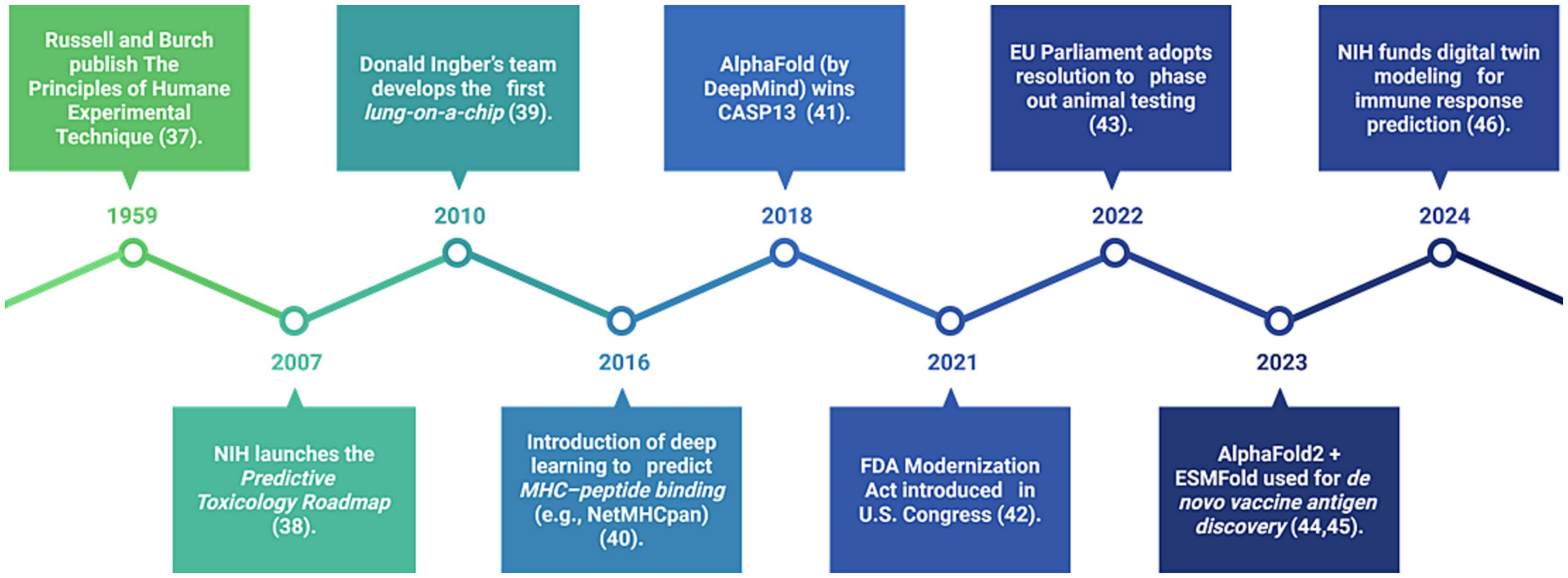
Timeline of key global milestones supporting AI and human-relevant alternatives in immunology (1959–2024). This figure illustrates the major ethical, scientific, and regulatory advancements that are accelerating the transition away from animal testing. It includes foundational efforts, the emergence of organ-on-a-chip technology, and AI-based immune prediction, as well as recent U.S. and European legislation promoting non-animal technologies in immunological research.

Artificial intelligence (AI) is rapidly transforming immunological research by enabling the simulation and analysis of complex immune responses. Machine learning algorithms trained on large, multimodal datasets, including single-cell RNA sequencing and real-world clinical outcomes, can now predict immune responses, identify vaccine targets, and classify disease subtypes with increasing precision. To better understand the trajectory of innovation driving the shift away from animal-based immunological research, we highlight key global developments in Figure 2. This visual timeline presents a curated series of milestones spanning from the foundational ethical framework to recent breakthroughs in artificial intelligence (AI)-driven modeling and regulatory reform. Events such as the NIH’s Predictive Toxicology Roadmap, the invention of organ-on-a-chip platforms, and the rise of deep learning tools like NetMHCpan and AlphaFold underscore the scientific and political momentum supporting the replacement of animal models. More recently, the use of AlphaFold2 and ESMFold for *de novo* vaccine antigen discovery, along with NIH’s funding of digital immune twins, marks a decisive turn toward AI-empowered, human-relevant platforms for immune modeling and vaccine design (Zhang et al., 2024; El Arab et al., 2025; Yang et al., 2023).

This review critically examines the role of AI in transforming pre-clinical immunology and vaccine development. We provide an overview of the technologies driving this shift, evaluate the scientific and regulatory challenges ahead, and argue for a balanced, hybrid approach that maximizes the strengths of computational, *in vitro*, and *in vivo* systems (Elfatimi et al., 2025; Xiao et al., 2025; Riviere et al., 2025; Alanazi, 2025; Barreto et al., 2025; Olawade et al., 2024; Omranian et al., 2024; Zhuang et al., 2024). By doing so, we aim to map a forward path that aligns scientific innovation with ethical responsibility and regulatory evolution.

In the following sections, we review (1) the scientific limitations of traditional animal models in immunology, (2) the role of AI in computational modeling and vaccine and immunotherapeutic designs, (3) emerging human-relevant alternatives such as organoids and organ-on-chip technologies (Sun et al., 2022; Farhang Doost and Srivastava, 2024; Alver et al., 2024; Wang et al., 2024; Picollet-D'hahan et al., 2021), (4) the economic and practical advantages of AI-driven methods, (5) regulatory and ethical frameworks, and (6) future directions for fully transitioning to AI-supported research pipelines.

## 2 Translational limitations of animal models in the development of vaccines and immunotherapeutics

Animal models have played a pivotal role in uncovering foundational concepts in immunology, including antigen recognition, immune cell trafficking, and cytokine signaling networks (Elfatimi et al., 2025; Reveiz et al., 2025; Chentoufi et al., 2025a; Chentoufi et al., 2025b; Quadiri et al., 2025b; Quadiri et al., 2025c; Srivastava et al., 2025; Vahed et al., 2025; Zayou et al., 2025). However, the translational reliability of these models in predicting human immune responses has become a growing concern within the scientific community. This concern arises from the fact that human and animal immune systems differ significantly in both structure and function, leading to inconsistencies in therapeutic outcomes when findings from pre-clinical animal studies are applied to clinical settings (Vunjak-Novakovic et al., 2021; Bailey, 2017). For example, laboratory mice, despite their widespread use in immunological research, exhibit distinct immune phenotypes shaped by their genetic homogeneity, controlled environments, and limited microbial diversity. These differences are manifested in altered immune cell distributions, varying cytokine secretion profiles, and divergent T-cell receptor repertoires compared to those of humans. As a result, immune therapies and vaccine candidates that show efficacy in mice frequently fail to reproduce the same effects in humans, particularly in diseases where immune modulation plays a central role, such as autoimmune disorders or chronic infections. Another challenge is that animal models often fail to adequately capture the complexity of human immune interactions across different organ systems. Immune responses in humans are shaped by a dynamic interplay among multiple tissue compartments, genetic backgrounds, environmental exposures, and microbiome factors, which are poorly replicated in traditional animal models. Additionally, specific immune processes, such as class-switch recombination in B cells or the formation of memory T cells, may proceed differently across species, further limiting the translational value of these models in vaccine development and the design of immunotherapies (Bailey, 2017).

These limitations have catalyzed a shift in focus toward more predictive and human-relevant systems. Immune organoids, developed from human lymphoid tissues, are a promising *in vitro* alternative by enabling the study of antigen-specific responses and germinal center dynamics. These 3D models preserve tissue architecture and cellular diversity, allowing the researchers to investigate vaccine-induced immunity under physiologically relevant conditions (Wagar et al., 2021; Wagoner et al., 2025; Prasad et al., 2021). In parallel, AI-based simulations offer computational models that can predict immune responses using patient-derived data, such as transcriptomic or single-cell sequencing profiles (Gui et al., 2023; Li, 2023; Li et al., 2024; Chambuso and Meena, 2025). These tools can simulate animal and human immune networks and forecast the outcomes of immunomodulatory interventions with increasing accuracy (Xu et al., 2023; Phongpreecha et al., 2025; Elfatimi et al., 2025). Nonetheless, these alternatives are not without their constraints. Human organoid models are often limited to single-organ contexts and lack systemic integration, whereas AI algorithms require robust and diverse datasets to ensure generalizability and accuracy (Fan et al., 2025; Kim et al., 2020; Huang et al., 2025; Liu X. et al., 2025). Despite their limitations, these emerging models present a compelling case for moving beyond traditional animal testing, especially when combined within hybrid experimental-computational frameworks that aim to preserve biological realism while enhancing predictive power.

To further contextualize the translational gap between animal models and human immunology, Table 1 presents a comparative overview of key immunological features distinguishing mouse models from the human immune system. These distinctions span cytokine expression, T-cell receptor diversity, microbiome complexity, MHC molecules, immune cell subsets, and exposure history. Each of these features plays a critical role in shaping immune responses, and their divergence underscores why findings in murine systems often fail to translate effectively to human clinical outcomes. This comparison highlights the urgent need for more predictive and human-relevant models in immunological research and therapeutic development.

**Table 1:** Comparative immunological features of mouse models versus the human immune system.

Feature	Mouse models	Human immune system
Cytokine expression	Distinct cytokine profiles: some cytokines (e.g., IL-8) are not naturally expressed (Du et al., 2017; Chow et al., 2024)	Broad, complex cytokine responses; IL-8 plays a key role in neutrophil recruitment
T-cell receptor (TCR) repertoire	Limited diversity due to inbred strains (Poltorak et al., 2018)	Highly diverse TCRs influenced by genetic and environmental factors
Microbiome influence	Lab-raised mice have limited microbial exposure (Hanski et al., 2024)	Human microbiota is more varied and shapes immune responses extensively
MHC molecules	Murine MHC genes differ significantly from HLA genes (Shiina et al., 2017)	The human leukocyte antigen (HLA) system is highly polymorphic and affects immunity
Immune cell subsets	Differences in NK cells, monocytes, and dendritic cell profiles (Parodi et al., 2023)	Broader variety and plasticity in immune cell subsets
Pathogen exposure history	Naïve immune systems in SPF conditions (Burger et al., 2023)	Humans have complex immune memory shaped by lifelong exposures

Highlights the key differences between commonly used mouse models and the human immune system, including cytokine expression, T-cell receptor repertoire, microbiome diversity, MHC molecules, immune cell subsets, and pathogen exposure history. These distinctions highlight the translational challenges of extrapolating findings on infection and immunity from animals to humans.

## 3 Artificial intelligence-powered models slash time and cost in vaccine and immunotherapeutic development

Artificial Intelligence (AI) is rapidly transforming immunology research by enabling advanced computational modeling and simulation approaches that reduce reliance on animal testing while improving the precision, speed, and scalability of vaccine development (Mak et al., 2014; Tang et al., 2019). With the complexity of human immune responses often poorly replicated in animal models, AI offers human-relevant alternatives by integrating large-scale biological data to simulate immune system dynamics, predict vaccine efficacy, and design novel immunization strategies (Yang et al., 2021; Ikram et al., 2023; Ito et al., 2024). AI-based systems use machine learning, deep learning, and systems biology approaches to model immune responses at the cellular and molecular levels (Sinicrope et al., 2024). These models can process multimodal datasets such as transcriptomics, proteomics, and imaging data to simulate antigen presentation, cytokine signaling, and immune memory formation *in silico* (Topol, 2019). For example, deep learning frameworks have been applied to predict B-cell and T-cell epitopes from protein sequences, improving the identification of potent antigenic targets for vaccine design without the need for animal immunization models (Jespersen et al., 2017). Additionally, reinforcement learning algorithms are being developed to de-risk vaccines and optimize dose scheduling and adjuvant selection based on simulated responses from a population (Shahzadi et al., 2024).

Virtual immune system platforms, such as C-ImmSim and agent-based models, enable detailed simulations of host-pathogen interactions, supporting hypothesis testing and comparative analysis of vaccine candidates before pre-clinical validation. These simulations have demonstrated their utility in evaluating the durability of immune protection and estimating population-wide outcomes in pandemic preparedness scenarios (Todman et al., 2008; Shinde et al., 2024). AI has also contributed to predicting adverse immune events, allowing early detection of potential reactogenicity based on immunological features, which would otherwise require lengthy and ethically concerning animal experiments (Kompa et al., 2022). Furthermore, integrating AI with human-relevant experimental platforms such as immune organoids, microfluidic systems, and organ-on-chip models creates a synergistic feedback loop (Sun et al., 2022; Farhang Doost and Srivastava, 2024; Alver et al., 2024; Wang et al., 2024; Picollet-D'hahan et al., 2021; Fan et al., 2025; Kim et al., 2020; Huang et al., 2025; Liu X. et al., 2025). These platforms provide high-fidelity data that trains and refines AI algorithms, leading to more accurate predictions of human immune responses (Gabriel et al., 2022; Ingber, 2022). Notably, recent studies have highlighted how AI-driven systems trained on human immune organoid data can outperform traditional models in forecasting vaccine outcomes (Fan et al., 2025; Kim et al., 2020; Huang et al., 2025; Liu X. et al., 2025; Morrocchi et al., 2024).

Another example of a platform is Vaxi-DL, a web-based deep learning server designed to predict potential vaccine and immunotherapy candidates using fully connected neural networks. Vaxi-DL models were trained on datasets containing antigenic and non-antigenic sequences derived from pathogens or cancers, as well as databases such as Protegen. The performance metrics reported include an average sensitivity of approximately 93%, accuracy, specificity, and the area under the ROC curve, demonstrating a good capability in correctly identifying protective antigens (or epitopes) across various pathogens and cancers. In comparison with other vaccine and immunotherapy prediction tools, such as Vaxign-ML and VaxiJen, Vaxi-DL performs well, often surpassing them in accuracy and efficiency, particularly in predicting positive vaccine and immunotherapy candidates. Vaxi-DL’s tool leverages extensive biological and physicochemical protein features for predictions, helping to prioritize candidates for further preclinical studies. It has been validated through performance metrics and benchmarking studies to be an effective deep learning tool for predicting vaccine and immunotherapy candidates, with high sensitivity and accuracy, thereby supporting its utility in accelerating the pre-clinical and clinical development of vaccines and immunotherapies. This case study presents concrete evidence of how AI can enhance candidate selection, minimize unnecessary animal experimentation, and contribute to cost-effective preclinical pipelines (Chang et al., 2025; Nierengarten, 2025; Dlamini et al., 2020; Villanueva-Meyer et al., 2024; Bakas et al., 2024).

**Figure 3:**
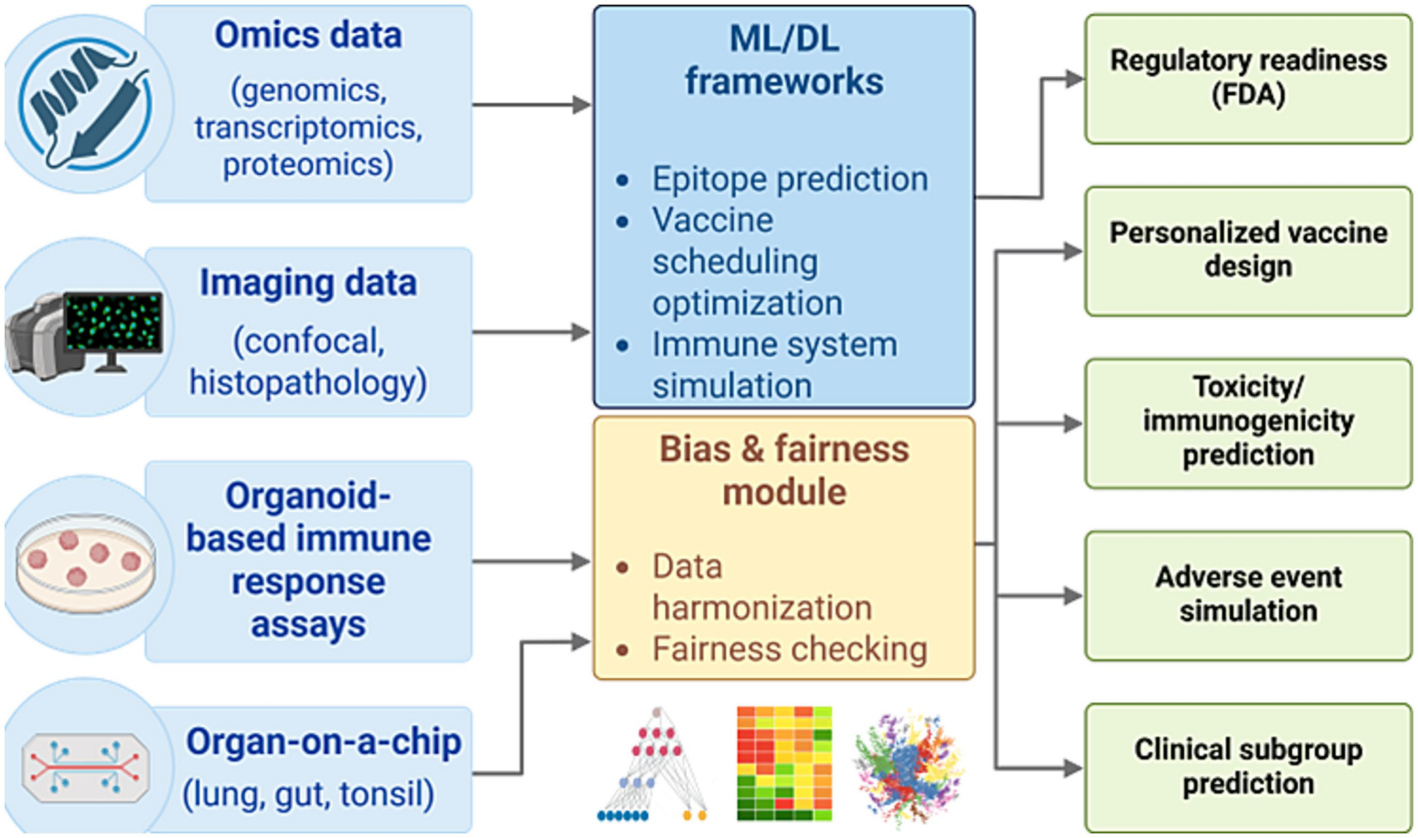
AI-human hybrid pipeline for ethical and predictive vaccine development. A conceptual workflow integrating omics, imaging, and experimental data with machine learning frameworks for simulating immune responses, optimizing vaccine design, and reducing reliance on animal models. The pipeline supports regulatory integration (FDA), ethical compliance, and human-relevant predictive outcomes across toxicity, efficacy, and subgroup analyses.

Recent advancements in computational immunology have led to the development of integrated AI-human hybrid pipelines that combine data from omics technologies, imaging, immune organoids, and organ-on-a-chip systems with machine learning frameworks (Fan et al., 2025; Kim et al., 2020; Huang et al., 2025; Liu X. et al., 2025; McGale et al., 2024). These systems enable *in silico* modeling of immune responses, epitope prediction, optimization of vaccine formulation, and bias-aware learning. Such pipelines not only enhance the speed and predictive accuracy of vaccine development but also significantly facilitate traditional animal testing. This approach supports regulatory readiness and ethical compliance by aligning with initiatives from the U.S. NIH and FDA to prioritize human-relevant methodologies (Nelson et al., 2024). As illustrated in Figure 3, this pipeline highlights the synergistic integration of multimodal data inputs, fairness-aware AI modules, and clinically actionable outputs that collectively transform the pre-clinical vaccine research landscape (Elfatimi et al., 2025; El Arab et al., 2025).

Overall, AI is expected to significantly advance pre-clinical animal studies in vaccines and immunotherapies by enhancing predictive modeling, optimizing experimental designs, and potentially reducing or replacing some animal experiments. AI and DL create computational models that predict B- and T-cell responses to vaccine and immunotherapy candidates more rapidly than traditional methods. For instance, NetMHCpan uses DL (artificial neural networks) to analyze peptide–MHC binding by training on large datasets with advanced encoding strategies for peptides and MHC molecules, improving the accuracy and generalizability of predictions (Phloyphisut et al., 2019). This helps identify optimal antigens or epitopes for improved vaccine and immunotherapy safety, efficacy, and durability. AI integrates multi-omics data and systems biology to phenotype and differentiate animal models B- and T-cell responses, allowing *in silico* testing of the safety, immunogenicity, and protective efficacy of vaccine and immunotherapy candidates without the need for extensive initial animal testing. AI enhances preclinical *in vivo* studies by optimizing study design and improving the translation of animal data to the clinic, increasing relevance and reproducibility (Deng and Strong, 2025; Han, 2025; Moassefi et al., 2023). AI-driven advanced modeling, combined with safety and efficacy simulations using digital twins and organ-on-chip platforms, simulates vaccine and immunotherapy safety in biological contexts, thereby further decreasing reliance on animal models (Sun et al., 2022; Farhang Doost and Srivastava, 2024; Alver et al., 2024; Wang et al., 2024; Picollet-D'hahan et al., 2021). These applications collectively enable faster, more ethical, and data-driven vaccine and immunotherapy development during preclinical phases by reducing or partly replacing animal experiments with AI-enhanced approaches (Imani et al., 2024). However, these computational predictions require validation against experimental data to ensure biological relevance and avoid overfitting or false positives.

Collectively, these innovations offer a paradigm shift toward ethical, efficient, and precise vaccine development pipelines. As computational power and biological data availability continue to expand, AI models in immunology are expected to become even more predictive and clinically actionable. Their ability to simulate personalized immune responses, forecast long-term protection, and guide next-generation vaccine strategies signals a future where AI replaces many functions previously performed through animal testing, ultimately leading to safer, faster, and more human-relevant biomedical research (Imani et al., 2024; Greener et al., 2022; Kumar et al., 2024).

## 4 Economic and practical benefits of AI in immunotherapy and vaccine research

In addition to scientific and ethical advances, artificial intelligence (AI) offers significant economic and logistical advantages over traditional animal models in immunology and vaccine development. Animal studies often require extensive financial and human resources, including specialized facilities, long-term animal care, and regulatory compliance infrastructure (Elfatimi et al., 2025; Rudroff, 2024; El Arab et al., 2025; Fu and Chen, 2025; Niu et al., 2025). The cumulative cost of these efforts contributes to the high price tag of drug and vaccine development, often exceeding $1 billion, with animal testing accounting for a substantial portion of pre-clinical R&D budgets (Fu and Chen, 2025; Van Norman, 2020; Van Norman, 2019; Acosta et al., 2011; Hasselgren and Oprea, 2024).

AI-based approaches provide a cost-effective alternative by enabling *in silico* simulations, drug screening, and immune modeling without the need for animal testing (Hasselgren and Oprea, 2024; Diogo Goncalves et al., 2025). Once foundational infrastructure, such as computational frameworks, data pipelines, and trained personnel, is established, AI tools can be rapidly reused and scaled at minimal marginal cost (Prathaban and Hande, 2024; Serrano et al., 2024). As illustrated in Table 2, AI is transforming every stage of the immunological research pipeline from early target identification to disease modeling by offering efficient, reproducible, and scalable alternatives to animal-based protocols (Deng and Strong, 2025; Han, 2025; Moassefi et al., 2023; Diogo Goncalves et al., 2025; Prathaban and Hande, 2024; Serrano et al., 2024; Lollini et al., 2006).

**Table 2:** Comparative framework of traditional animal-based research versus AI-driven approaches in pre-clinical development of drugs, vaccines, and immunotherapeutics.

Research purpose	Traditional animal-based methods	AI-driven approaches
Drug/vaccine/immunotherapeutic screenings	*In vivo* challenge tests, immunogenicity, protective efficacy, immune assays Toxicity studies in rodents and non-human primates	AI-guided virtual screening of compounds *In silico* simulation of immune activation and toxicity
Target discovery	Gene knockout and transgenic animal studies Observational findings from animal pathology	AI analysis of omics data (genomics, transcriptomics, proteomics) Pattern discovery in large datasets
Lead optimization	Medicinal chemistry adjusted based on animal response Dose–response curves in animals	Predictive modeling of pharmacodynamics and ADMET Machine learning for epitope-antigen matching
Efficacy evaluation	Behavioral and clinical scoring in animal disease models Tissue pathology and serology	Simulation of immune protection *in silico* Clinical trial data used for predictive efficacy modeling
Safety and toxicity	Long-term exposure studies in multiple species Organ-specific toxicity observed post-mortem	AI prediction of adverse events from molecular structure Toxicogenomic-based machine learning tools
Pharmacokinetics (PK)	ADME and bioavailability tracking via labeled substances Tissue sampling at set intervals	AI-based modeling of drug distribution PBPK models derived from real-world and simulated data
Biomarker identification	Cytokine profiling and immune markers in animal fluids Histological scoring	AI integration of multi-omics for biomarker prediction Deep learning to identify signature pathways
Disease modeling	Animal induction of disease *via* infection or mutation Progression tracked over weeks/months	AI simulation of immune dysregulation Longitudinal modeling of patient-derived datasets

Comparison of conventional *in vivo* methods with artificial intelligence (AI)-powered strategies across the significant stages in drug, vaccine, and immunotherapeutic development. This includes toxicity, immunogenicity, and protective screening, target discovery, lead optimization, efficacy evaluation, safety and toxicity assessment, pharmacokinetics, biomarker identification, and disease modeling.

For example, AI-driven epitope prediction and virtual antigen screening platforms can analyze millions of antigen-target combinations within hours, compared to the weeks or months required for *in vivo* testing (Diogo Goncalves et al., 2025; Eshak and Goupil-Lamy, 2025; Eshak et al., 2024). This accelerated timeline not only reduces development cycles but also minimizes the number of failed candidates entering clinical trials. Recent estimates suggest that integrating AI into drug discovery pipelines can reduce the total development cost by 50–70% and decrease the time-to-market by several years (Rudroff, 2024; Fu and Chen, 2025; Diogo Goncalves et al., 2025; Vora et al., 2023; March et al., 2025; Arora et al., 2024; Gangwal and Lavecchia, 2025). Moreover, AI systems facilitate parallel processing and rapid iteration, allowing researchers to test multiple hypotheses simultaneously, which would be impractical with live animal models (Hasselgren and Oprea, 2024; Diogo Goncalves et al., 2025). Platforms such as DeepMind’s AlphaFold2 or immune organoid-AI hybrids have already demonstrated success in structure-based antigen prediction, epitope mapping, and toxicity forecasting (Fan et al., 2025; Kim et al., 2020; Huang et al., 2025; Liu X. et al., 2025; Diogo Goncalves et al., 2025). This flexibility is especially valuable in pandemic situations or when addressing emerging pathogens, where time is a critical factor (Rudroff, 2024; El Arab et al., 2025; Fu and Chen, 2025; Gangwal and Lavecchia, 2025).

The transition to AI-powered platforms also allows for more sustainable allocation of research funding. No widely standardized percentage of a university’s total research infrastructure budget is devoted to animal facilities (Holbrook and Sanberg, 2013). However, it has been estimated that up to 15–20% of a university’s research infrastructure budget is dedicated to the upkeep of animal research facilities, highlighting a significant area where costs could be redirected toward AI-driven tools and computational resources (Holbrook and Sanberg, 2013). A growing number of institutions are beginning to reallocate investments from animal housing and breeding facilities to computational resources and personnel specializing in data science (Gangwal and Lavecchia, 2025; He et al., 2025; Ajisafe et al., 2025; Mehta et al., 2025), representing a substantial financial burden that could be redirected toward AI infrastructure. This shift not only promotes long-term cost efficiency but also enhances reproducibility and transparency (Deng and Strong, 2025; Han, 2025; Moassefi et al., 2023). AI models can be easily shared, versioned, and audited, unlike animal studies, which often suffer from variability, irreproducibility, and inter-laboratory inconsistencies (Deng and Strong, 2025; Han, 2025; Moassefi et al., 2023). However, the reproducibility of AI results also depends on transparent reporting of model architectures, training data availability, and computing environments, which are now increasingly standardized through open-source frameworks and FAIR data principles (Deng and Strong, 2025; Han, 2025; Moassefi et al., 2023).

Furthermore, AI supports real-time learning and adaptation. As new biological data, whether from clinical trials, organ-on-chip platforms, or immune organoids, become available, models can be retrained and improved without having to restart the experimental process (Sun et al., 2022; Farhang Doost and Srivastava, 2024; Alver et al., 2024; Wang et al., 2024; Picollet-D'hahan et al., 2021; Fan et al., 2025; Kim et al., 2020; Huang et al., 2025; Liu X. et al., 2025; Niazi and Mariam, 2025). This dynamic feedback loop accelerates the optimization of vaccine candidates and immune modulators, ultimately driving faster regulatory approval and broader accessibility.

In summary, AI represents not only a scientific innovation but also a paradigm shift in the economics and efficiency of immunological research (Elfatimi et al., 2025; El Arab et al., 2025; Goktas and Damadoglu, 2025). By reducing costs, increasing scalability, and eliminating many practical barriers of animal experimentation, AI lays the foundation for a more ethical, rapid, and data-driven future in vaccine development and immunotherapy research.

## 5 Emergent AI and human-relevant alternatives in vaccine and immunotherapeutic development

From both scientific and ethical standpoints, there is an urgent need to reimagine pre-clinical immunology using methods that are more human-relevant and technologically scalable (Elfatimi et al., 2025; Alanazi, 2025). Over the past two decades, a paradigm shift has emerged in pre-clinical and translational research, driven by increasing challenges and limitations of animal models and a parallel surge in computational and tissue engineering technologies.

In response to these challenges, major regulatory bodies have institutionalized this shift. In 2010, the European Directive 2010/63/EU (1) provided a framework for animal testing through the three R’s: Replacement, Reduction, and Refinement; and (2) formulated the ultimate goal of entirely replacing animal experiments with non-animal methods when scientifically possible, marking the beginning of the phase-out process of animal experimentation in the EU. More recently, the U.S. Food and Drug Administration (FDA) and the National Institutes of Health (NIH) have launched landmark initiatives to reduce and eventually eliminate the requirement for animal testing in the development of biologics, including vaccines (Elfatimi et al., 2025; Xiao et al., 2025; Riviere et al., 2025; Alanazi, 2025; Barreto et al., 2025; Olawade et al., 2024; Omranian et al., 2024; Zhuang et al., 2024). These agencies are encouraging the adoption of advanced human-based methodologies, including artificial intelligence (AI), human-derived organoids, organ-on-a-chip platforms, and induced pluripotent stem cell (iPSC) technologies (Alver et al., 2024; Nelson et al., 2024; Ouyang et al., 2019). These models aim to capture the nuances of human immune physiology in a more accurate and ethically acceptable manner.

Artificial intelligence has been applied to model host–pathogen interactions, performing virtual high-throughput screening of vaccine candidates, and simulating immune dynamics under various therapeutic conditions. These applications are already reshaping how immunological questions are framed and answered (Xu et al., 2023; Bender and Cortes-Ciriano, 2021; Goldberg and Hartung, 2006; Hartung, 2010; Vinken et al., 2021; Rawal et al., 2022). For instance, AI-based models integrated multi-omics datasets to simulate B- and T-cell responses, cytokine and chemokine signaling, and antigen presentation, offering immunologically relevant representations that improve upon traditional static models (Elfatimi et al., 2025; Farzan, 2024; Sharma et al., 2022). These models enable the real-time tracking of B- and T-cell responses, including T-cell function, T-cell exhaustion, and cross-reactivity, allowing for the optimization of vaccine and immunotherapeutic designs (Elfatimi et al., 2025; Kumar et al., 2024). In another example, AI facilitates reverse vaccinology, epitope prediction, and personalized vaccine and immunotherapeutic formulation by integrating large-scale immunological data (Elfatimi et al., 2025; Farzan, 2024; Sharma et al., 2022; Ito et al., 2024; Kumar et al., 2024). This reduces time and costs by filtering out ineffective vaccine and immunotherapeutic candidates before pre-clinical and clinical trials. In both animal models and humans, AI can predict the B- and T-cell responses to vaccines and immunotherapies, allowing dynamic adjustments and refinement of immunological interpretations (Elfatimi et al., 2025; Farzan, 2024). It enhances predictions of cytokine and chemokine networks and immune checkpoint dynamics, thereby contributing to the development of more effective vaccines and immunotherapeutics (Elfatimi et al., 2025; Farzan, 2024; Sharma et al., 2022). The U.S. NIH has recently established the Office of Research Innovation, Validation, and Application (ORIVA), which is tasked with developing and validating non-animal-based models across the NIH’s research portfolio (Goktas and Grzybowski, 2025). This marked a clear move toward prioritizing “human-relevant” technologies that could bridge the gap between laboratory science and real-world human biology, particularly in areas such as immunology and vaccine development.

Among these alternatives, artificial intelligence (AI) stands out as a transformative tool for rethinking how immune responses are modeled and predicted. Deep learning and machine learning algorithms are now being widely employed for epitope prediction, vaccine antigen optimization, immune repertoire classification, and simulation of host-pathogen interactions (Xu et al., 2023; Sinicrope et al., 2024; Chen et al., 2023). For example, AI-based methods can analyze massive immunological datasets, such as those from flow cytometry, RNA-seq, proteomics, and clinical trials, to identify novel antigenic targets or anticipate adverse immune reactions. These algorithms are capable of uncovering hidden patterns in human data that would be difficult to detect using traditional statistical methods, enabling more personalized and predictive vaccine design (Kumar et al., 2024). Simultaneously, the FDA has released a regulatory roadmap to begin reducing reliance on animal testing for biologics, including monoclonal antibodies and vaccine candidates. The FDA’s policy encourages the integration of computational simulations, immune organoids, and organ-on-a-chip technologies into pre-clinical pipelines over the next 5 years, aiming to make animal studies the exception rather than the norm (Fan et al., 2025; Kim et al., 2020; Huang et al., 2025; Liu X. et al., 2025; Nelson et al., 2024). These changes are reinforced by a growing ecosystem of public-private partnerships and research consortia focused on AI-powered precision medicine, including applications in immunotoxicity, vaccine durability, and the prediction of immune escape.

Human immune organoids have emerged as another promising platform for research (Fan et al., 2025; Kim et al., 2020; Huang et al., 2025; Liu X. et al., 2025). These 3D miniaturized lymphoid tissues, often derived from human tonsils, spleens, or iPSC-derived immune progenitors, have been shown to replicate essential features of adaptive immunity, including germinal center formation, antibody class switching, and T–B cell interactions (Wagar et al., 2021; Kastenschmidt et al., 2023). These features make them particularly attractive for vaccine evaluation, as they allow scientists to study antigen-specific responses *in vitro* under near-physiological conditions. When combined with AI-based analytical pipelines, organoids enable more efficient hypothesis generation, screening, and mechanistic insight, all without relying on animals (Fan et al., 2025; Kim et al., 2020; Huang et al., 2025; Liu X. et al., 2025).

Organ-on-a-chip (OoC) systems offer yet another level of complexity. These microfluidic devices mimic the dynamic biochemical, mechanical, and cellular microenvironments of human tissues. Immunology-focused OoCs can model lung, gut, or skin immune barriers, supporting studies on mucosal immunity, vaccine delivery, and adjuvant response. For example, Ingber and colleagues have developed multiorgan chip systems that enable the real-time analysis of interactions between immune cells and target tissues to be studied in real-time (Nithin et al., 2023; Tian et al., 2024). When used in conjunction with AI models, these systems can generate multiscale simulations that incorporate tissue-specific immune responses, pharmacokinetics, and safety profiles. Despite the enormous potential of these technologies, significant limitations remain. Most current organoid and chip-based systems simulate responses in isolated compartments and do not replicate the systemic coordination observed in complete immune responses, an essential feature for understanding vaccine-induced protection or immune-related adverse events (Fan et al., 2025; Kim et al., 2020; Huang et al., 2025; Liu X. et al., 2025). Moreover, many AI models lack sufficient external validation, and their performance can degrade when applied to new populations or unseen clinical scenarios due to dataset biases or limited training diversity (Huang et al., 2022). However, some of the organoids and organ-on-chips are not strictly human-based and may still incorporate animal-derived components or cells (Sun et al., 2022; Farhang Doost and Srivastava, 2024; Alver et al., 2024; Wang et al., 2024; Picollet-D'hahan et al., 2021; Fan et al., 2025; Kim et al., 2020; Huang et al., 2025; Liu X. et al., 2025; Ingber, 2022; Liu K. et al., 2025; Horejs, 2021). Despite these limitations, the field is progressing toward increasingly human-relevant models, aiming to reduce animal use by more accurately mimicking human physiology (Ingber, 2022; Liu K. et al., 2025). While the transition to completely human-based systems is ongoing, challenges remain in fully replicating complex tissue environments and functions without incorporating animal elements (Elfatimi et al., 2025; Xiao et al., 2025; Riviere et al., 2025; Alanazi, 2025; Barreto et al., 2025; Olawade et al., 2024; Omranian et al., 2024; Zhuang et al., 2024; Ingber, 2022; Liu K. et al., 2025; Horejs, 2021).

Integrative approaches that combine organoids, organ-on-chip (OoC) platforms, and artificial intelligence (AI) are being developed to overcome these shortcomings (Sun et al., 2022; Farhang Doost and Srivastava, 2024; Alver et al., 2024; Wang et al., 2024; Picollet-D'hahan et al., 2021; Fan et al., 2025; Kim et al., 2020; Huang et al., 2025; Liu X. et al., 2025). These multiscale models aim to reconstruct both cellular-level interactions and system-wide immune responses, but they are still in their infancy. Additional regulatory guidelines, benchmarking standards, and collaborative infrastructures are necessary to facilitate the routine adoption of these approaches in immunological research and vaccine development pipelines.

Table 3 provides an overview of emerging human-relevant technologies designed to reduce reliance on animal models in immunological research. It highlights key platforms, including artificial intelligence (AI), immune organoids, organ-on-a-chip systems, and integrated multiscale models, alongside their primary features, applications in immunology, and current limitations (Fan et al., 2025; Kim et al., 2020; Huang et al., 2025; Liu X. et al., 2025). The table also includes the NIH’s ORIVA program, which supports the validation and adoption of these alternatives. Collectively, these tools offer promising avenues for modeling immune responses more accurately and ethically, however, challenges such as limited systemic integration and scalability still need to be addressed (Elfatimi et al., 2025; Xiao et al., 2025; Riviere et al., 2025; Alanazi, 2025; Barreto et al., 2025; Olawade et al., 2024; Omranian et al., 2024; Zhuang et al., 2024).

**Table 3:** Emerging human-relevant alternatives to animal models in immunology (Singer and Akhtar, 2024).

Platform/tool	Key features	Applications in immunology	Limitations
Artificial intelligence (AI)	Predictive modeling using large datasets (e.g., transcriptomics, EHR, imaging) (Singer and Akhtar, 2024; Diray-Arce et al., 2022)	Epitope prediction, antigen design, immune simulation, vaccine response forecast	Depends on dataset quality; limited generalizability across populations
Immune organoids	3D lymphoid tissues derived from tonsil, spleen, or iPSCs (Braham et al., 2023)	Study of germinal center reactions, T-B cell interaction, and antibody production	Limited to single-organ systems; lacks systemic integration
Organ-on-a-chip (OoC)	Microfluidic devices that mimic human tissue architecture and microenvironments (Sinha et al., 2018)	Barrier immunity (e.g., gut, lung), vaccine delivery, and immunotoxicity studies	High complexity; expensive; limited scalability across complete immune systems
Multiscale hybrid models	Integration of AI + Organoids + OoC for systemic simulation (Morrison et al., 2024; Suhito et al., 2025)	Comprehensive simulation of immune response across compartments	Still in development; no regulatory standardization yet
ORIVA (NIH Program)	NIH office dedicated to the validation of non-animal research models	Funding and coordinating the adoption of human-relevant tools	Early-stage implementation; depends on interagency and interinstitutional support

Summary of innovative AI platforms, immune organoids, organ-on-a-chip systems, multiscale hybrid models, and the NIH ORIVA initiative. Key features of vaccine and immunotherapeutic applications, as well as current limitations, are outlined, illustrating the growing ecosystem of non-animal, human-relevant research tools.

## 6 Benefits of AI in pre-clinical vaccine and immunotherapeutic development

The current practices in animal models used in research always consider the “three Rs”: (1) Replacement involves assessing the scientific justification and rationale for using an animal in biomedical research, including whether there are alternatives to using live animals. (2) Reduction entails assessing whether the number of animals required in an experiment is adequate to achieve scientifically valid results. This includes power and statistical tests to determine the smallest number of animals in each experiment that would be sufficient to produce statistically meaningful results. (3) Refinement will determine whether and how animal procedures are likely to cause pain or distress, and how this can be minimized. This involves ensuring that similar animal experiments are not already reported in the scientific literature and describing the potential novelty of the additional experiment.

**Figure 4:**
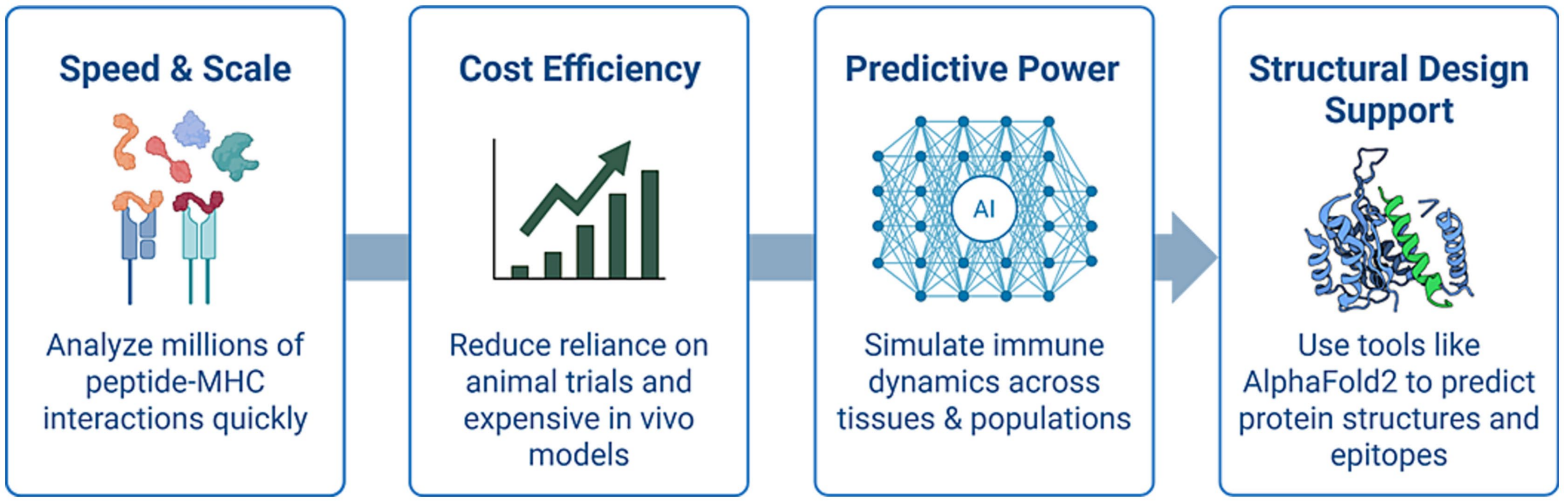
Benefits of AI in pre-clinical immunological research. Key advantages of artificial intelligence (AI) in pre-clinical immunological research: AI technologies enhance the speed and scalability of screening peptide–MHC interactions, reduce reliance on costly and ethically challenging animal models, provide predictive insights into immune responses across diverse human populations, and support structure-based immunogen design using tools like AlphaFold2.

Artificial Intelligence (AI), which has rapidly become a critical tool in reshaping the landscape of pre-clinical immunological research, will certainly help accelerate the implementation of the “three Rs” above (Rudroff, 2024). AI offers numerous advantages over traditional animal-based models, providing both scientific and ethical advancements in how we investigate immune responses and design therapeutic interventions by leveraging high-throughput data and complex algorithms. Figure 4 provides a visual summary of the key advantages of AI in pre-clinical immunology, including improved speed, predictive power, cost-efficiency, structural insight, and ethical impact.

### 6.1 Speed and scale

AI enables researchers to analyze millions of potential peptide MHC interactions *in silico* within hours. This task would be impractical using animal experiments or conventional laboratory techniques. This rapid computational screening significantly accelerates the prioritization of vaccine targets and epitope candidates. Deep neural networks trained on human immunopeptidome data can predict binding affinities and T-cell immunogenicity with high precision, thereby narrowing down the most promising antigens for experimental validation (Xu et al., 2023; Phloyphisut et al., 2019).

### 6.2 Cost efficiency

AI-driven platforms have already demonstrated substantial cost savings in drug discovery pipelines, particularly in oncology (Hasselgren and Oprea, 2024; Vora et al., 2023). Similar frameworks are now being adapted for the treatment of infectious diseases. By de-risking vaccine and immunotherapeutic discoveries and reducing reliance on expensive and time-consuming *in vivo* studies, AI enables pre-clinical development teams to conduct early-stage hypothesis testing, virtual compound screening, and immunological modeling at a fraction of the cost of animal-based trials (Gangwal and Lavecchia, 2025; Bonaiti et al., 2024). Additionally, these efficiencies can help reduce financial barriers for smaller research institutions and startups working in global vaccine development.

While AI-driven pre-clinical assessments of vaccines and immunotherapeutic candidates can reduce animal use and lower expenses, the extent to which AI benefits the economy by lowering the cost of pre-clinical animal experiments for vaccines and immunotherapy candidates remains to be determined. For instance, AI-powered drug discovery platforms are expected to reduce expenses associated with pre-clinical animal studies by 50–70%. Overall, AI has the potential to save millions of dollars and years compared to traditional animal-based experiments to select safe, immunogenic, and protective vaccine and immunotherapy candidates (Chang et al., 2025). Recent return on investment analyses indicate that AI applications in immunotherapy are accelerating cost savings (Chang et al., 2025; Nierengarten, 2025; Villanueva-Meyer et al., 2024; Bakas et al., 2024). AI-driven tools, including deep learning models integrated with digital pathology, imaging, and multi-omic datasets, are enabling more intelligent resource allocation by targeting therapies to patients most likely to respond, reducing waste in clinical trials, and decreasing unnecessary treatments (Chang et al., 2025; Nierengarten, 2025; Dlamini et al., 2020).

### 6.3 Predictive power

Unlike animal models, which often fail to replicate human immune responses accurately, AI systems can be trained on real-world datasets, including single-cell transcriptomics, clinical trial data, and immune repertoire profiles, to simulate immune dynamics across diverse human populations. These simulations can be used to predict immune responses to novel antigens, identify patient subgroups most likely to benefit from a given vaccine or immunotherapy, and model rare adverse events such as cytokine storms or immune escape (Xu et al., 2023; Kastenschmidt et al., 2023). This level of predictive insight is not possible in animal systems due to their biological and immunological constraints.

### 6.4 Ethical soundness

One of the most compelling arguments for the adoption of AI in pre-clinical animal research is its potential to drastically reduce, and eventually eliminate, the use of animal subjects. Immunogenicity testing, toxicity screening, and inflammation profiling can now be partially or fully modeled through AI simulations and human-based organoid systems (Fan et al., 2025; Kim et al., 2020; Huang et al., 2025; Liu X. et al., 2025). This shift not only aligns with the enhanced principles (Replacement, Reduction, Refinement) but also improves public trust and acceptance in biomedical research by promoting more humane and sustainable practices (Rudroff, 2024; Ingber, 2022; Nithin et al., 2023).

### 6.5 Technological synergy with structural prediction tools

AI models such as AlphaFold2 have revolutionized structural biology by predicting 3D protein folding with near-experimental accuracy. This capability is highly valuable in vaccine design, where structural information on viral antigens or immune receptors is critical for epitope mapping and rational immunogen selection. Combined with machine learning algorithms tailored to accelerate and de-risk epitope discovery and antigenicity scoring, these technologies are streamlining the conceptualization and optimization of immune intervention (Gangwal and Lavecchia, 2025; Tian et al., 2024).

Overall, AI represents a scalable, ethical, and scientifically superior alternative for many aspects of immunological research. When integrated with complementary technologies, such as organoids and organ-on-chip systems, AI has the potential to create fully human-relevant pipelines that replace traditional animal models in both discovery and translational immunology (Sun et al., 2022; Farhang Doost and Srivastava, 2024; Alver et al., 2024; Wang et al., 2024; Picollet-D'hahan et al., 2021; Fan et al., 2025; Kim et al., 2020; Huang et al., 2025; Liu X. et al., 2025).

## 7 Challenges and limitations

While artificial intelligence (AI) offers immense promise in reducing and replacing animal models in immunology, several limitations must still be acknowledged (Elfatimi et al., 2025; Xiao et al., 2025; Riviere et al., 2025; Alanazi, 2025; Barreto et al., 2025; Olawade et al., 2024; Omranian et al., 2024; Zhuang et al., 2024). Despite their increasing sophistication, current AI models still cannot fully replicate the complexity of a living organism. Certain conditions, particularly systemic immune disorders, and long-term physiological responses, remain challenging to model without *in vivo* studies (Rudroff, 2024; Kamimoto et al., 2023; Laurent et al., 2024; Blanc et al., 2025).

Several scientific barriers to entirely replacing animal studies with AI stem from the complexity of living systems and the current limitations of computational models (Rudroff, 2024; Kamimoto et al., 2023; Fruhwein and Paul, 2025). For instance, AI is less effective at discovering new side effects, toxicity pathways, or immunological mechanisms that emerge *in vivo* following the administration of vaccine and immunotherapeutic candidates, particularly those that are not represented in the initial AI training data (Rudroff, 2024; Kamimoto et al., 2023). Some immune responses only appear in the context of a functioning organism as a whole and therefore are difficult to predict through AI simulations alone (Rudroff, 2024; Kamimoto et al., 2023). Moreover, ethical replacement of animal studies by AI should proceed with a commitment to validate and implement human-relevant models, grounded not only in animal welfare but also in science’s duty to generate reliable and translatable data for human health (Fruhwein and Paul, 2025).

A key concern is the risk of bias in training data. Suppose AI models are developed using datasets that primarily reflect a narrow demographic. In that case, they may produce less accurate or even misleading predictions for other populations, including women, children, or older adults (El Arab et al., 2025). Addressing such biases requires deliberate efforts to diversify and balance input data during model training and validation. Complex datasets from genomics and proteomics may yield false-positive correlations due to various factors, such as data size, technical variability, and statistical noise (Lafit et al., 2019; Huttlin et al., 2007; Zhang et al., 2015). For instance, errors in identifying protein antigens to be incorporated into vaccine and immunotherapeutic candidates as differentially expressed in proteomics (false positives) are common and require careful experimental design and statistical testing *in vitro* and in animals to avoid misinterpretation (Lafit et al., 2019; Huttlin et al., 2007; Zhang et al., 2015). This highlights the importance of using robust statistical approaches, high-quality data preprocessing, and critical biological validation to ensure that identified correlations are both biologically plausible and actionable (Huttlin et al., 2007; Zhang et al., 2015). Thus, without rigorous validation—such as biochemical confirmation, replication in comparable biological contexts, and advanced statistical controls—false-positive correlations in genomics and proteomics can mislead vaccine and immunotherapeutic research, as well as clinical decision-making (Lafit et al., 2019; Huttlin et al., 2007; Zhang et al., 2015).

Overfitting is another technical limitation, where models may perform well on internal datasets but fail to generalize to external or unseen data (Yang et al., 2023). This can lead to overly optimistic performance estimates and reduced real-world applicability. To mitigate this, rigorous validation methods such as cross-validation, regularization techniques, and external benchmarking are essential during model development.

High-dimensional biomedical data, including omics and imaging datasets, can also increase the risk of spurious associations or biologically implausible outputs (Xiao et al., 2025; Omranian et al., 2024). Researchers must therefore interpret AI-generated predictions cautiously and prioritize experimental validation, especially in applications involving the development of vaccines or immunotherapies.

Other limitations of AI include (1) the “black-box” problem (Duran and Jongsma, 2021; Rudin, 2019) and (2) the complex process of model retraining with new data (Rudin, 2019; Vela et al., 2022). (1) The black-box problem refers to the lack of transparency in how complex AI and deep learning models arrive at their decisions (Sinicrope et al., 2024; Duran and Jongsma, 2021; Rudin, 2019; Vela et al., 2022). These models learn from vast amounts of data and develop intricate internal representations that human natural intelligence, including the model designers, often cannot fully understand or explain (Duran and Jongsma, 2021; Rudin, 2019; Vela et al., 2022). This opacity makes it difficult to trace or interpret the decision-making process, creating issues with trust, accountability, and the ethical use of resources (Duran and Jongsma, 2021; Rudin, 2019; Vela et al., 2022). (2) Retraining AI models with new data is a time-consuming and laborious process (Vela et al., 2022). It typically requires large volumes of labeled data and careful tuning to avoid issues such as “catastrophic forgetting” (where a model loses previously acquired knowledge), lack of convergence, and variability due to random initialization. Automating frequent retraining is challenging and can introduce significant risks (Vela et al., 2022). In clinical and translational applications, retraining often necessitates additional regulatory review, documentation, and validation before deployment, thereby further increasing the time and cost burden. These limitations underscore fundamental difficulties in making AI systems trustworthy, reliable, and responsive to new data while maintaining transparency and clarity that are understandable to human natural intelligence (Vela et al., 2022).

Despite its transformative potential, AI is not a panacea. Most models are only as reliable as the data on which they are trained, and many suffer from inherent biases tied to demographics, geography, or time (Prathaban and Hande, 2024). Moreover, while organoid models offer valuable alternatives to animal tissues, current systems such as those derived from tonsils often reflect isolated lymphoid structures and fail to reproduce the complex, systemic immune interactions that occur during real infections or vaccinations (Deng and Strong, 2025; Han, 2025; Moassefi et al., 2023; Wagar et al., 2021; Fan et al., 2025; Kim et al., 2020; Huang et al., 2025; Liu X. et al., 2025; Kastenschmidt et al., 2023). As such, AI-based predictions still require experimental validation, and moving from animal-based to fully digital pipelines demand standardization, benchmarking, and ongoing refinement (Shahabipour et al., 2023). Safety concerns remain particularly relevant, as even the most advanced AI systems may not yet fully capture toxicological or immunopathological risks, making final *in vivo* validation necessary in many cases (Wagar et al., 2021; Bar-Ephraim et al., 2020). Moreover, many AI techniques, intensive learning still function as “black boxes,” where the underlying reasoning behind predictions is challenging to interpret (Riviere et al., 2025). This lack of transparency can limit trust among clinicians, immunologists, and regulators. Ongoing advancements in explainable AI aim to address this challenge, but wide-scale adoption remains limited.

There are also practical challenges to implementing AI approaches (Elfatimi et al., 2025; Xiao et al., 2025; Riviere et al., 2025; Alanazi, 2025; Barreto et al., 2025; Olawade et al., 2024; Omranian et al., 2024; Zhuang et al., 2024). Some research institutions may lack access to computational infrastructure, specialized personnel, or funding required to utilize AI-based tools fully. Additionally, regulatory bodies are still adapting their frameworks to evaluate and approve AI-generated evidence, which means that even well-performing models may face barriers to clinical integration.

While AI faces significant challenges related to data biases, there are established approaches to address them effectively (Elfatimi et al., 2025; Xiao et al., 2025; Riviere et al., 2025; Alanazi, 2025; Barreto et al., 2025; Olawade et al., 2024; Omranian et al., 2024; Zhuang et al., 2024). Among the challenges is that a bias can stem from unbalanced datasets, flawed data collection processes, or inherited societal prejudices within the data (Elfatimi et al., 2025; Xiao et al., 2025; Riviere et al., 2025; Alanazi, 2025; Barreto et al., 2025; Olawade et al., 2024; Omranian et al., 2024; Zhuang et al., 2024). This bias can lead to reduced fairness, trust issues, and ethical concerns, impacting pre-clinical and clinical decision-making for vaccines and immunotherapies. A major contributing factor is the difficulty of acquiring large, well-curated, and standardized multi-omic datasets from diverse human populations. Such data collection efforts are expensive, time-consuming, and complicated by privacy regulations and a lack of harmonized data formats across institutions, which limit data sharing and the generalizability of models. This issue can be mitigated by (1) cleaning, balancing, and transforming data to reduce discrimination before training; (2) implementing fairness constraints, counterfactual fairness, and re-weighting to ensure equitable decision outcomes across demographics; (3) calibrating model outputs to improve fairness after decisions are made; and (4) implementing human oversight for continuous bias auditing and transparent reporting of AI decision logic.

There also exist challenges related to deep learning networks, which operate as “black boxes” where the decision-making process is unclear, thereby hindering trust, regulatory compliance, and bias detection (Elfatimi et al., 2025; Xiao et al., 2025; Riviere et al., 2025; Alanazi, 2025; Barreto et al., 2025; Olawade et al., 2024; Omranian et al., 2024; Zhuang et al., 2024). This can also be mitigated by (1) using interpretable models or applying post-hoc explanation tools like LIME (Local Interpretable Model-Agnostic Explanations) and SHAP (SHapley Additive exPlanations) to explain which features influence decisions; (2) providing transparency through documentation of model training, data characteristics, and decision logic fosters stakeholder understanding and trust; (3) encouraging human-in-the-loop frameworks that allow experts to validate AI decisions and improve system outcomes; and (4) aligning with regulatory requirements for transparency and explainability, particularly in industries requiring high accountability. The challenges of data biases and interpretability in AI are addressable through a multi-faceted approach involving technical solutions, organizational practices, transparency, and collaboration, resulting in fairer, more reliable, and trustworthy AI systems (Elfatimi et al., 2025; Xiao et al., 2025; Riviere et al., 2025; Alanazi, 2025; Barreto et al., 2025; Olawade et al., 2024; Omranian et al., 2024; Zhuang et al., 2024).

Another challenge with AI is that data biases in digital immune twins affect predictive reliability (Weinberger et al., 2025). These biases typically arise because the datasets used to train these predictive models often overrepresent populations with specific ethnicities, races, and genders, as well as genetic mix-ups (e.g., males, whites, affluent individuals), and underrepresent others (e.g., women, non-binary individuals, marginalized groups) (Weinberger et al., 2025). This imbalance in data leads to algorithmic biases that impact the accuracy and fairness of model predictions for underrepresented groups, potentially reducing the overall reliability of these models in pre-clinical and clinical decision-making for vaccines and immunotherapies (Mann, 2024; De Domenico et al., 2025). Specifically, the predictive reliability of digital immune twins suffers when the underlying data lacks representation of physiological, demographic, or socio-medical diversity (Weinberger et al., 2025). This can result in inaccurate or less effective vaccine and immunotherapy predictions, as well as disease progression simulations, for specific populations. Such limitations are especially critical when simulating systemic immune responses, for example, systemic inflammation or long-term protection, where missing population diversity can lead to misleading projections of efficacy or adverse events. Structural exclusions in data and limited diversity in training datasets exacerbate these issues, reinforcing health disparities rather than mitigating them (Mann, 2024; De Domenico et al., 2025).

Furthermore, these biases in digital immune twins are not merely theoretical concerns: AI models trained on biased data tend to propagate and even amplify these biases, resulting in less trustworthy predictions and decision-making for vaccines and immunotherapies in pre-clinical and clinical settings (Mann, 2024; De Domenico et al., 2025). Efforts to detect, understand, and correct biases using simulations or bias correction algorithms exist, but challenges remain to eliminate prejudice and improve model reliability for all populations entirely. In summary, data biases in digital immune twins significantly affect their predictive reliability, especially when those biases lead to underrepresentation of key patient subgroups, impacting both the accuracy and equity of predictions (Weinberger et al., 2025; Mann, 2024; De Domenico et al., 2025).

Despite these hurdles, the scientific, economic, and ethical motivations for adopting non-animal approaches are gaining traction. Traditional animal models are increasingly viewed as limited in their ability to predict human outcomes, costly to maintain, and misaligned with public sentiment regarding animal welfare. Consequently, many leading research institutions and pharmaceutical companies are beginning the shift toward AI-based, human-relevant systems.

**Figure 5:**
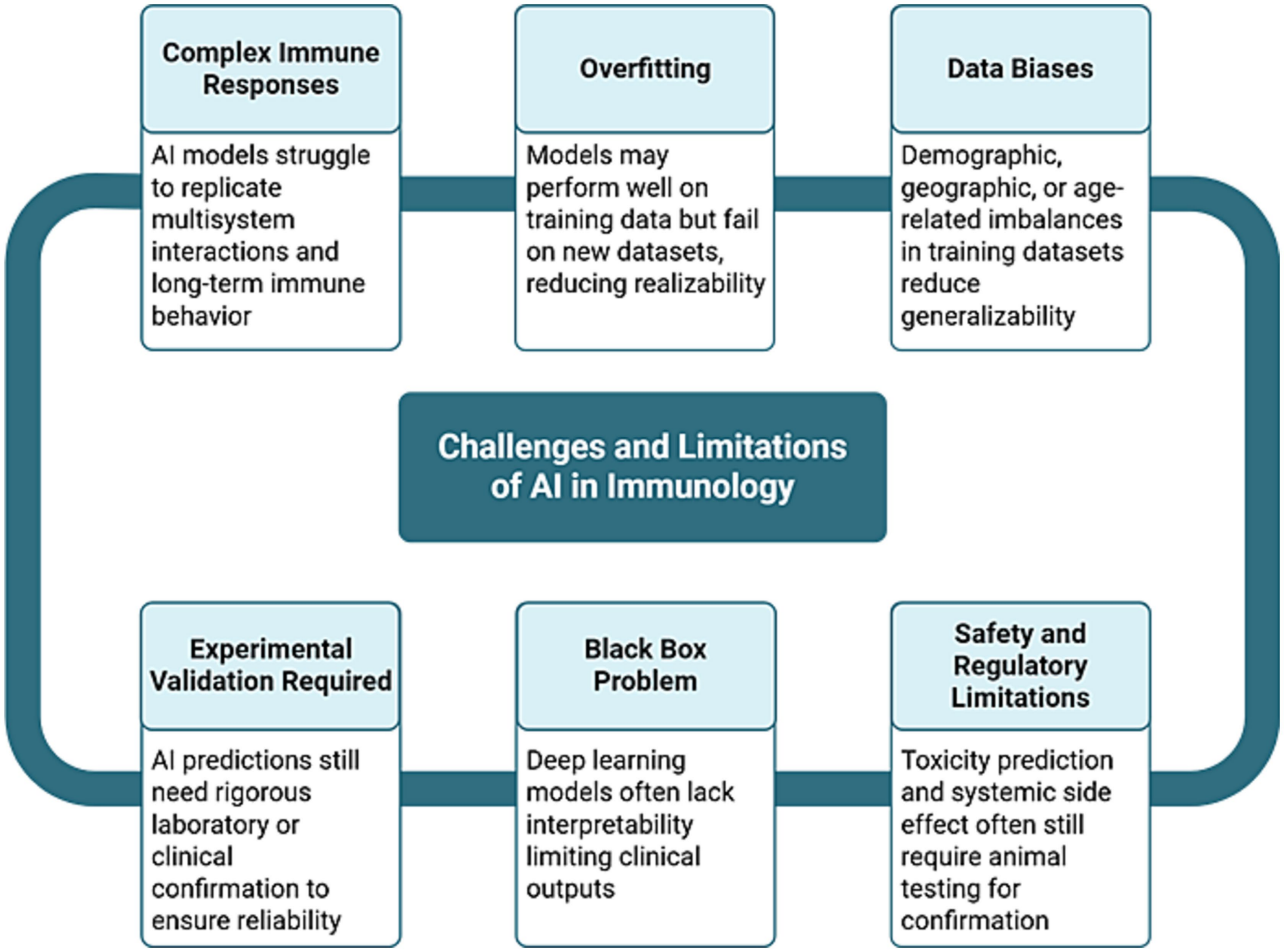
Challenges and limitations of AI in immunology. This flowchart summarizes the key obstacles that limit the complete replacement of animal models in immunology with AI (Elfatimi et al., 2025; Xiao et al., 2025; Riviere et al., 2025; Alanazi, 2025; Barreto et al., 2025; Olawade et al., 2024; Omranian et al., 2024; Zhuang et al., 2024). These include complex immune responses that are challenging to model, data biases that reduce generalizability, overfitting on training data, the lack of interpretability in deep learning models (the “black box problem”), the ongoing need for experimental validation, and the continued reliance on animal testing for confirming toxicity and safety. Each challenge is interconnected, highlighting the need for a multidisciplinary and standardized approach to overcome these challenges (Elfatimi et al., 2025; Xiao et al., 2025; Riviere et al., 2025; Alanazi, 2025; Barreto et al., 2025; Olawade et al., 2024; Omranian et al., 2024; Zhuang et al., 2024).

While AI may not immediately replace animal models in every context, it is likely to progressively reduce reliance on them, first by supporting early-stage screening and hypothesis generation, and eventually by enabling comprehensive immune modeling pipelines. As AI systems continue to mature and align with clinical data, they are expected to become both more trustworthy and scientifically superior. Rather than an abrupt end to animal research, this will represent a gradual but inevitable evolution toward more ethical, accurate, and efficient biomedical science. Figure 5 provides a visual summary of the key challenges discussed in this section, illustrating how limitations such as data bias, model overfitting, interpretability issues, and the need for experimental validation remain critical barriers to replacing animal models with AI-driven approaches in immunology (Elfatimi et al., 2025; Xiao et al., 2025; Riviere et al., 2025; Alanazi, 2025; Barreto et al., 2025; Olawade et al., 2024; Omranian et al., 2024; Zhuang et al., 2024).

## 8 Regulatory momentum and funding initiatives

Encouragingly, regulatory bodies are now actively supporting the shift. The NIH has begun prioritizing research projects that avoid animal testing and explicitly encourages “human-relevant” methodologies (Nelson et al., 2024). The FDA similarly states that animal testing should become the exception, not the norm (Nelson et al., 2024). In a landmark decision reflecting a growing commitment to ethical research practices, the NIH announced the creation of the Office of Research Innovation, Validation, and Application (ORIVA). This new office is tasked with promoting and funding non-animal research methods, marking a significant shift towards human-relevant science. The initiative is hailed as a major victory for animal ethics, aligning scientific research with ethical imperatives and public sentiment. ORIVA’s mission includes scaling AI and organoid models across immunological research domains (Fan et al., 2025; Kim et al., 2020; Huang et al., 2025; Liu X. et al., 2025). However, for this transformation to succeed, dedicated funding is required for researcher training, infrastructure development, and cross-laboratory standardization (Wagar, 2023; Kastenschmidt et al., 2023). Beyond technical limitations, the persistence of animal methods bias in scientific publishing and research funding has also been identified as a significant barrier to the adoption of non-animal alternatives. Kavanagh and Krebs highlighted how entrenched preferences for animal models, despite the availability of human-relevant tools, can hinder scientific progress and misallocate research resources (Nelson et al., 2024). Addressing such biases through policy reform, reviewer education, and structural changes in grant evaluation is essential to support the broader implementation of AI-integrated, organoid, and organ-on-chip-based platforms (Alver et al., 2024). By prioritizing these alternative methods, the NIH aims to enhance the relevance and applicability of biomedical research to human health outcomes (Sun et al., 2022; Farhang Doost and Srivastava, 2024; Alver et al., 2024; Wang et al., 2024; Picollet-D'hahan et al., 2021; Fan et al., 2025; Kim et al., 2020; Huang et al., 2025; Liu X. et al., 2025; Nelson et al., 2024).

## 9 Future of AI-based approaches to aid animal model experiments in vaccine and immunotherapeutic development

The path toward reducing and eventually replacing animal models in immunology and vaccine research is increasingly shaped by the rapid evolution of artificial intelligence (AI), human-relevant models, and cross-disciplinary innovation (Elfatimi et al., 2025; El Arab et al., 2025; Goktas and Damadoglu, 2025). This transformation is no longer a speculative goal; it is unfolding now through advances in AI-driven modeling, regulatory support, and integration of biological data across scales (Elfatimi et al., 2025; El Arab et al., 2025; Goktas and Damadoglu, 2025).

Several international efforts have already laid the groundwork for this transition. Initiatives such as the NIH’s ORIVA program and similar regulatory developments in the U.S. and Europe are accelerating the validation and adoption of AI-based platforms in immunological research. The U.S. FDA has recently initiated efforts to eliminate specific animal testing requirements, promoting the use of AI-driven computational models and human organoid platforms to strengthen pre-clinical assessments and increase their relevance to human biology (Fan et al., 2025; Kim et al., 2020; Huang et al., 2025; Liu X. et al., 2025). The FDA Modernization Act 2.0 marks a pivotal shift by officially endorsing non-animal methods such as AI-driven platforms as valid tools for specific pre-clinical assessments (Elfatimi et al., 2025; Rawal et al., 2022; Preeti et al., 2023). Among these innovations, systems like Vaxi-DL, a deep learning framework for vaccine antigen prediction, illustrate how *in silico* approaches can accurately prioritize high-potential candidates, substantially decreasing reliance on animal experiments (Elfatimi et al., 2025; Rawal et al., 2022; Preeti et al., 2023). In addition, emerging AI models now possess the capability to simulate key pharmacological processes, including pharmacokinetics, metabolic pathways, and immune system interactions, enabling rapid and efficient virtual screening of therapeutic agents before human trials (Elfatimi et al., 2025; Rawal et al., 2022; Preeti et al., 2023). These developments are being matched by growing investment from biotech companies, startups, and public-private consortia committed to developing non-animal testing strategies for immune-related diseases and vaccine evaluation.

One of the most exciting areas of progress is the development of multiscale, system-level AI models that can simulate complex immune responses. Unlike earlier models focused on isolated molecular events, new AI frameworks are incorporating data from genomics, proteomics, transcriptomics, imaging, and clinical outcomes to build integrated, patient-specific simulations. These tools enable researchers to model immune pathways *in silico* with increasing biological realism, thereby drastically reducing the need for animal experimentation.

Equally transformative is the rise of *in silico* clinical trials. These virtual trials use AI to simulate how different individuals might respond to vaccines or immunotherapies based on their immune profile, potentially reducing reliance on both pre-clinical animal testing and early-stage human trials. As regulatory agencies begin to develop clear frameworks for validating AI-generated evidence, we expect these simulations to become a standard component of ethical vaccine development pipelines. Another key priority is AI explainability, which refers to the ability of models to provide interpretable predictions that can be trusted by immunologists, clinicians, and regulators. Progress in this area will increase transparency and help shift AI from a mere analytical tool to a collaborative partner in experimental design, hypothesis generation, and clinical translation.

Emerging computational tools, such as neuromorphic systems and digital immune twins, personalized virtual representations of a patient’s immune system, promise to enhance modeling precision further, making it possible to test immunotherapies and vaccines entirely *in silico* under realistic, individualized conditions (Kumar et al., 2024). In diseases such as autoimmune disorders and chronic infections, where animal models have repeatedly failed to predict human outcomes, AI-driven insights may accelerate and de-risk the discovery of new therapeutic targets and biomarkers (Gangwal and Lavecchia, 2025). This could unlock breakthrough treatments in areas that have long stagnated due to the limitations of traditional animal-based approaches. Ultimately, the expansion of AI in immunology must be guided by clear and established ethical standards. As we entrust machines with decisions that influence human health, building frameworks for fairness, transparency, and responsible data use is not an option; it is essential.

To successfully transition from traditional animal models to human-relevant research tools, the field of immunology must adopt a multifaceted strategy that integrates advanced technologies, regulatory frameworks, and ethical considerations. Artificial intelligence (AI) plays a central role in this shift, offering scalable, predictive, and ethically sound alternatives for modeling immune responses and evaluating vaccine efficacy. Table 4 outlines the key innovation areas driving this transformation, detailing the current limitations, anticipated advancements, and expected impact of AI across various domains of immunological research.

**Table 4:** Strategic advances in artificial intelligence to replace animal models in pre-clinical development of drugs, vaccines, and immunotherapeutics.

Focus area	Current limitations	Future outlook	Expected impact
AI model complexity	Focused on narrow immune mechanisms	Development of multiscale, systems-level immune models	Better prediction of immunotherapy outcomes and vaccine efficacy
Data integration	Fragmented omics and clinical datasets	Seamless fusion of genomic, proteomic, and patient data	Personalized immune modeling and biomarker discovery
Virtual trials for predicting immune response	Used only in simple simulation scenarios	Expansion to simulate diverse immune responses across populations	Ethical, rapid, and cost-effective pre-clinical testing
Regulatory validation of AI-driven pre-clinical tools	Limited formal validation of AI tools	Regulatory frameworks for AI validation in immunology research	Faster approval and integration of non-animal methods
AI explainability	Many models remain “black boxes”	Transparent, interpretable AI models	Enhanced trust, reproducibility, and collaboration across fields
AI–human collaboration	AI used as a supplementary analytical tool	AI as a proactive partner in hypothesis generation and experiment design	Accelerated discovery with improved scientific rigor
Digital immune twins	Conceptual and limited to research use	Fully functional, patient-specific immune simulations	Tailored vaccine design and immune therapy optimization
AI in autoimmune/chronic disease	Traditional models poorly replicate human immune pathologies	AI-driven discovery of disease mechanisms and response profiles	New treatments in hard-to-model immune conditions
Ethical frameworks for AI Use	Ethical guidelines remain emergent	Establishment of responsible standards for AI in immunological research	Trustworthy and human-centric development of digital biomedical tools

Current limitations, future outlooks, and expected impacts of AI-driven innovations across multiple domains are outlined. This includes model complexity, data integration, virtual trials, regulatory validation, explainability, AI-human collaboration, digital immune twins, modeling of autoimmune/chronic diseases, and ethical frameworks.

Future directions of AI and quantum computing in simulating complex molecular interactions for vaccine and immunotherapy development in animal models will focus on several key advances and impacts (Elfatimi et al., 2025; Contreras et al., 2022; de la Fuente et al., 2022; de la Fuente and Contreras, 2023). AI and DL will continue to actively transform vaccine and immunotherapy research through predictive frameworks that enable rapid, data-driven decision-making and the integration of multi-omics data with computational models (Elfatimi et al., 2025; Contreras et al., 2022; de la Fuente et al., 2022; de la Fuente and Contreras, 2023). This will include better phenotyping and classification of diseases, as well as tailored vaccine and immunotherapy designs and refined antigen/epitope selections to enhance the efficacy and durability of immune protection (Elfatimi et al., 2025). AI is expected to push further toward the potential replacement of traditional animal preclinical testing with computational simulations, as supported by initiatives from the NIH and FDA to phase out some animal testing in favor of AI models (Elfatimi et al., 2025). AI will uncover more complex immune interactions not evident through traditional experimental assays, helping to guide novel vaccine and immunotherapeutic strategies, improve the precision of immune response predictions, and optimize vaccine and immunotherapeutic formulations (Elfatimi et al., 2025). Advanced AI techniques, such as generative models, multimodal learning, and interpretable machine learning, will further accelerate the design of personalized vaccines and immunotherapies, enabling the simulation and optimization of immune responses *in silico* before animal or human trials (Elfatimi et al., 2025; Kumar et al., 2024).

Additionally, emerging approaches such as federated learning enable model training across decentralized datasets from multiple institutions without sharing raw data, thereby preserving patient privacy while improving model generalizability and reducing bias, an essential step for building robust vaccine prediction pipelines. Quantum computing is expected to simulate the molecular interactions and complex vaccine and immunotherapeutic molecules with unprecedented precision, surpassing the limits of classical computation (Elfatimi et al., 2025; Contreras et al., 2022; de la Fuente et al., 2022; de la Fuente and Contreras, 2023). This capability will enhance the prediction of interactions, efficacy, safety, pharmacodynamics, and toxicity of vaccine and immunotherapeutic molecules (Elfatimi et al., 2025; Contreras et al., 2022; de la Fuente et al., 2022; de la Fuente and Contreras, 2023). By improving the accuracy of vaccine and immunotherapeutic molecule simulations, quantum computing will support the reduction of reliance on animal testing, the refinement of lead compounds more efficiently, and the prediction of toxicity earlier in pre-clinical development (Elfatimi et al., 2025; Contreras et al., 2022; de la Fuente et al., 2022; de la Fuente and Contreras, 2023). Quantum-enhanced AI and quantum machine learning optimize molecular dynamics simulations for antigen and epitope discovery, protein folding, and elucidation of immune system mechanisms, which are critical to vaccine and immunotherapy development (Elfatimi et al., 2025; Contreras et al., 2022; de la Fuente et al., 2022; de la Fuente and Contreras, 2023). Quantum vaccinomics will integrate quantum computing with immunogenetics and genomics to identify protective immune antigens and epitopes, and design vaccines and immunotherapeutic molecules. This approach will utilize both *in silico* and experimental methods to elucidate protective immune mechanisms (Elfatimi et al., 2025; Contreras et al., 2022; de la Fuente et al., 2022; de la Fuente and Contreras, 2023). Thus, the synergy of AI and quantum computing is expected to significantly accelerate the design, testing, and optimization of vaccines and immunotherapies by providing deep insights into immune regulation and disease mechanisms (Elfatimi et al., 2025; Contreras et al., 2022; de la Fuente et al., 2022; de la Fuente and Contreras, 2023). This combination is expected to enhance translational relevance by delivering more accurate computational models that can replace animal models in many preclinical contexts, thereby reducing ethical concerns and development times (Elfatimi et al., 2025; Contreras et al., 2022; de la Fuente et al., 2022; de la Fuente and Contreras, 2023). Regulatory agencies are increasingly recognizing and supporting these transformative computational technologies to modernize and improve preclinical evaluation pipelines (Elfatimi et al., 2025; Contreras et al., 2022; de la Fuente et al., 2022; de la Fuente and Contreras, 2023).

Finally, another direction of preclinical animal studies is moving towards Hybrid Intelligence (HI), which combines Natural Intelligence (NI) and Artificial Intelligence (AI) (Sollini et al., 2020; Jia et al., 2022; Saravi et al., 2022; Kurvers et al., 2023; Hirosawa et al., 2024). On one hand, the NI spans the breadth of human (and collective) cognition, emotion, and ethical understanding, encompassing not only individual thinking but also group dynamics, societal norms, and planetary well-being. It reflects our ability to empathize, innovate, sense our surroundings, and collaborate at every level (Loaiza et al., 2024; Mohite et al., 2024; Ou et al., 2025). On the other hand, AI encompasses computational systems and algorithms designed to process large datasets, discern patterns, and handle tasks—such as language understanding or predictive analytics—that traditionally rely on human-like intelligence (Imani et al., 2024; Huemer et al., 2020; Natali et al., 2021; Liu et al., 2024; Qin et al., 2024; Zakariya et al., 2024; Bhujel et al., 2025; Kedar et al., 2025; Zhao and Wang, 2025). The combination provides HI, which represents the synergy between AI’s speed and analytical rigor, and NI’s depth of insight. Researchers in animal models harness solid, data-driven capabilities by uniting both, while still honoring essential human values, ethical reasoning, and collective stewardship (Zhao and Wang, 2025; Hou et al., 2024; Zemelka-Wiacek et al., 2024; Lv et al., 2025; Zhou et al., 2025).

## 10 Conclusion

The integration of artificial intelligence (AI) into biomedical research represents a transformative opportunity to improve experiments in animal models, particularly in immunology and the development of vaccines and immunotherapies (Xiao et al., 2025). AI-driven approaches such as deep learning, mechanistic modeling, digital immune twins, and *in silico* clinical trials offer the potential to simulate complex immune responses with increasing precision, speed, and ethical accountability. When combined with advanced technologies like organoids, organ-on-a-chip systems, and multi-omics integration, these tools can model human-relevant biology in a more scalable and predictive manner than traditional animal experiments (Fan et al., 2025; Kim et al., 2020; Huang et al., 2025; Liu X. et al., 2025).

On one hand, animal models remain critical for biological research due to their complex physiology and living-system context, which current AI and *in vitro* methods cannot fully replace (Chang and Grieder, 2024; Barre-Sinoussi and Montagutelli, 2015). Animal models facilitate an understanding of whole-organism biology, development, and systemic responses that AI simulations, based on existing data and algorithms, may not yet fully capture (Barre-Sinoussi and Montagutelli, 2015). On the other hand, AI advancements are increasingly supporting the refinement and reduction of animal use by automating immune response assessments, screening vaccine and immunotherapy candidates, and improving the precision of *in vivo* experiments (Alanazi, 2025; Germain et al., 2024; Gururaj et al., 2024). Comparisons between AI models and animal models reveal both complementarities and distinctions. AI models, including machine learning (ML) and deep learning (DL), provide powerful tools for optimizing preclinical animal experiments to test vaccine and immunotherapy candidates by analyzing complex datasets, enhancing experimental design, predicting outcomes, and extracting more meaningful insights from experiments (Germain et al., 2024; Gururaj et al., 2024). AI can also integrate results from animal models with human clinical data to improve the translational relevance of vaccine and immunotherapy candidates. AI-based methods are scalable, adaptable, and can run simulations much faster than animal experiments, often in hours or days instead of weeks or months. This enables the rapid exploration of multiple hypotheses without the ethical and resource burdens associated with breeding, housing, and handling animals. In summary, AI and animal models serve partly overlapping but distinct functions: AI enhances, complements, and can partially substitute for animal experiments, especially in data analysis, simulation, and rapid hypothesis testing. However, animal models remain indispensable for capturing complex biological systems *in vivo*, pending further development of AI (Germain et al., 2024; Gururaj et al., 2024).

While AI, organoids, and organ-on-a-chip systems are rapidly transforming immunological research, they cannot yet fully replace animal models (Fan et al., 2025; Kim et al., 2020; Huang et al., 2025; Liu X. et al., 2025). Multiorgan immune interactions, long-term vaccine responses, and real-world safety evaluations still require *in vivo* validation. The future lies not in the immediate elimination of animal research but in progressively reducing dependence on it through validated, high-fidelity digital and *in vitro* alternatives (Elfatimi et al., 2025; El Arab et al., 2025; Goktas and Damadoglu, 2025). Rather than marking the end of animal testing, the rise of AI in immunology should be seen as the beginning of a more advanced and ethical form of biomedicine. This transition supports the development of scalable, human-relevant solutions in vaccine and immunotherapy research.

Despite the promise of these technologies, several challenges remain. Issues such as data bias, overfitting, limited model explainability, and the need for experimental validation must be addressed through standardized workflows, transparent reporting, and cooperative regulatory development (Elfatimi et al., 2025; Xiao et al., 2025; Riviere et al., 2025; Alanazi, 2025; Barreto et al., 2025; Olawade et al., 2024; Omranian et al., 2024; Zhuang et al., 2024). AI should be viewed as a gradual solution that first reduces animal use by accelerating and de-risking early-stage discovery, and eventually advances to replace more complex applications as the technology improves (Gangwal and Lavecchia, 2025).

Regulatory agencies, such as the U.S. Food and Drug Administration, have already begun to recognize AI-based tools as valid alternatives for specific pre-clinical evaluations. Large-scale initiatives, including the National Institutes of Health’s Office of Research and Infrastructure Programs and ORIVA, support the development and validation of these methods. Increasing public awareness, economic efficiency, and scientific demand for human-relevant models are all contributing to this shift in research strategy. In the long term, the responsible and collaborative use of AI, grounded in human biology and aligned with ethical frameworks, will shape the next generation of biomedical innovation. The future will not be defined by a binary choice between animal and digital models. Instead, it will emerge from a synergistic ecosystem where computational tools refine, augment, and ultimately replace animal testing (Elfatimi et al., 2025; El Arab et al., 2025; Goktas and Damadoglu, 2025). This evolution holds the potential to accelerate and de-risk the discovery of drugs, vaccines, and immunotherapies while upholding the highest standards of ethical and scientific integrity.

Equally transformative is the integration of AI with advanced experimental systems such as three-dimensional (3D) immune organoids and organ-on-a-chip (OoC) technologies (Fan et al., 2025; Kim et al., 2020; Huang et al., 2025; Liu X. et al., 2025). Human immune organoids, derived from secondary lymphoid tissues such as tonsils, have been shown to replicate critical elements of adaptive immunity, including germinal center formation and isotype switching (Wagar et al., 2021; Fan et al., 2025; Kim et al., 2020; Huang et al., 2025; Liu X. et al., 2025; Kastenschmidt et al., 2023; Morales Pantoja et al., 2023; Smirnova et al., 2023). When combined with computational models, these platforms enable detailed study of immune memory, evaluation of vaccine formats, and screening of adjuvants, all without relying on animal models. Similarly, OoC technologies, pioneered by researchers such as Donald Ingber, simulate vascular and epithelial–immune interactions across multiple organ systems, providing insight into systemic immune responses that single-organ models cannot capture (Ingber, 2022; Ingber, 2006).

As AI continues to evolve in complexity, interpretability, and ethical oversight, it is poised to redefine how we model immune responses, evaluate vaccines, and develop immunotherapies, ushering in a new era of precision medicine with less reliance on animals and greater alignment with human biology.
